# P-1037. Prevalence and antifungal susceptibility of *Candida* species isolated from Korean infants and children with candidemia, 2011-2023

**DOI:** 10.1093/ofid/ofae631.1227

**Published:** 2025-01-29

**Authors:** Hye Jeong Moon, Da Yun Kang, Youngmin Cho, Hyunju Lee, Eun Hwa Choi, Ki Wook Yun

**Affiliations:** Seoul National University Children's Hospital, Seoul, Seoul-t'ukpyolsi, Republic of Korea; Department of Pediatrics, Seoul National University Children’s Hospital, Seoul, South Korea, seoul, Seoul-t'ukpyolsi, Republic of Korea; Seoul National University Bundang Hospital, Seongnam-si, Kyonggi-do, Republic of Korea; Seoul National University Bundang Hospital, Seongnam-si, Kyonggi-do, Republic of Korea; Seoul National University Children's Hospital, Seoul, Seoul-t'ukpyolsi, Republic of Korea; Seoul National University Children's Hospital, Seoul, Seoul-t'ukpyolsi, Republic of Korea

## Abstract

**Background:**

Candida is the most common cause of fungal infections, and candidemia is the most common form of invasive fungal infection. The widespread use of antifungal agents has led to a global increase in non-albicans Candida infections and antifungal resistance. This study aims to investigate the clinical characteristics, treatment outcomes, fungal species, and antifungal susceptibility results of candidemia cases in Korean children.

Trend of annual candidemia prevalence and distribution of Candida species

**Figure:**
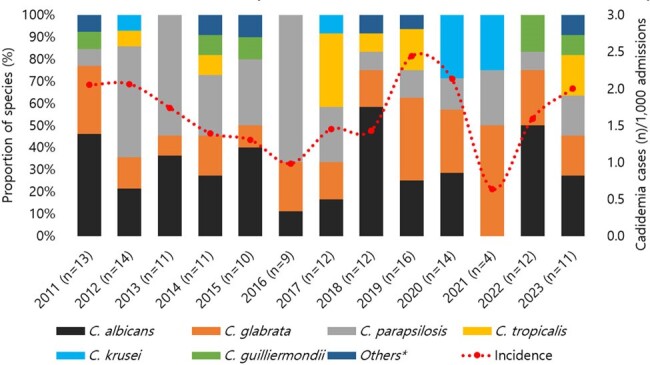

**Methods:**

This study retrospectively collected and analyzed clinical data from Korean children at two referring hospitals from 2011 to 2023. A case of candidemia was defined as a positive confirmation of any *Candida* species on at least one blood culture. Information on the *Candida* species and antifungal susceptibility was obtained from the results of the VITEK II automated equipment. The annual incidence of candidemia was adjusted to the combined number of hospitalized children in the two hospitals, and statistical analysis was performed on 30-day mortality as risk factors.

Distribution of Candida species according to the age groups

**Figure:**
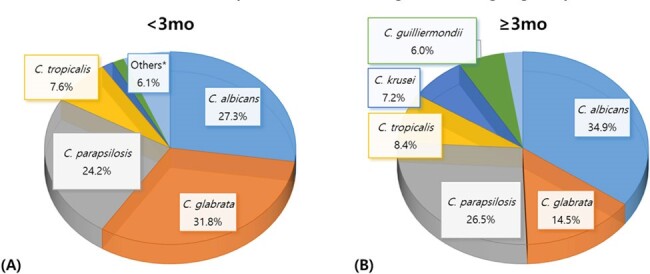

**Results:**

A total of 144 cases of candidemia were diagnosed in 139 children. The prevalence rate of candidemia per 1,000 actual hospitalized children was 0.6-2.4 (median 1.6). The age distribution showed 46.0% of individuals were younger than three months. All patients had an underlying medical condition, with prematurity (93.2%) most common in those under three months and hemato-oncology (HO) disease (44.0%) in those three months and older. In the younger age group, *Candida glabrata* was the most prevalent (31.8%), followed by *Candida albicans* (27.3%) and *Candida parapsilosis* (24.2%). In the older age group, *C. albicans* was the most prevalent (34.9%), followed by *C. parapsilosis* (26.5%). Non-susceptibility were 0.9% for amphotericin, 15.6% for fluconazole, 0% for voriconazole, 2.9% for caspofungin, and 7.7% for micafungin. The 30-day mortality rate for candidemia was 28%. The distribution of the *Candida* species was comparable between the mortality and survival groups. Age of 5-9 years and HO underlying disease were significantly associated with 30-day mortality.

Antifungal non-susceptibility according to Candida species

**Figure:**
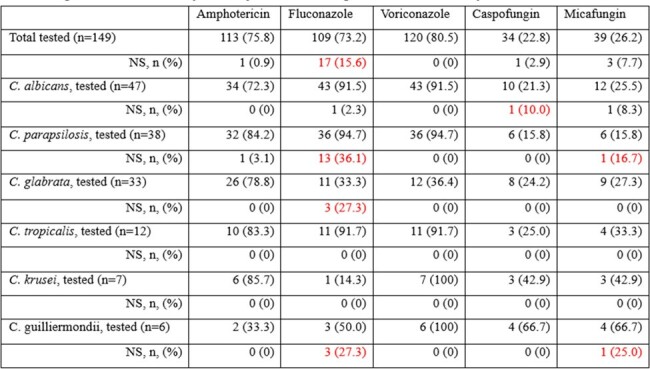

**Conclusion:**

The proportion of candidemia caused by non-albicans *Candida* species remained high in Korean children, requiring individualized strategies for initial treatment based on age group and underlying medical conditions.

Risk factors for 30-mortality in Korean children with candidemia

**Figure:**
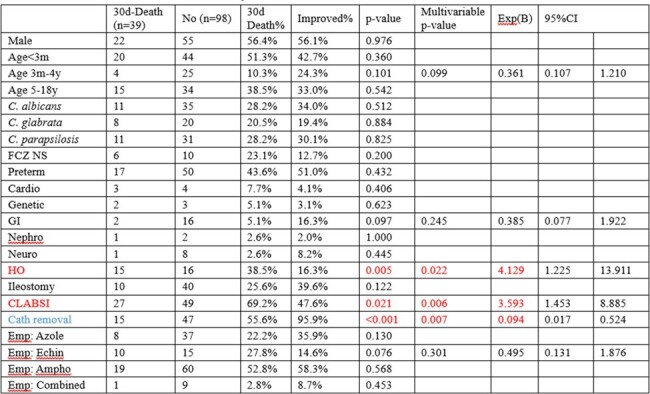

**Disclosures:**

**All Authors**: No reported disclosures

